# Endovascular treatment for basilar trunk artery aneurysm in the flow diverter era: a consecutive series and review of literature

**DOI:** 10.1186/s41016-025-00422-6

**Published:** 2026-03-05

**Authors:** Hengwei Jin, Jian Lv, Wei You, Xinke Liu, Hongwei He, Wei Feng, Youxiang Li

**Affiliations:** 1 Department of Neurosurgery, Beijing Tiantan Hospital, Capital Medical University, No.119, South 4 Ring West Road, Fengtai District, Beijing, 100070 China; 2 Department of Interventional Neuroradiology, Beijing Neurosurgical Institute, No.119, South 4 Ring West Road, Fengtai District, Beijing, 100070 China; 3 Department of Neurosurgery, Songyuan Jilin Oilfield Hospital, No.960, Yan Jiang Xi Road, Songyuan, Jilin, 138001 China

**Keywords:** Basilar trunk aneurysm, Endovascular treatment

## Abstract

**Background:**

Endovascular treatment (EVT) for basilar artery trunk aneurysm (BTA) is inadequately studied due to its rarity. The aim of this study was to report our experience regarding treatment modalities, complications and outcomes.

**Methods:**

A retrospective analysis of 2759 aneurysm patients who underwent EVT between January 2018 and December 2022 was performed. Patients with BTAs were involved, and their clinical characteristics, treatment modalities, complications, and clinical and angiographic outcomes were collected. Literatures from 2013 to 2024 were reviewed and studies included more than 5 BTA cases were summarized.

**Results:**

Thirty-seven patients were involved, including 6 (16.2%) patients with ruptured BTAs. Treatment modalities included simple coiling for 5 (13.5%) patients, traditional low-metal-coverage stent for 1 (2.7%) patient, stent-assisted coiling for 20 (54.1%) patients, and flow diverter (FD) for 11 (29.7%) patients. Four (10.8%) procedure-related complications occurred, including 1 (2.7%) hemorrhage and 3 (8.1%) ischemia cases. The last angiographic follow-up (mean 9.5 ± 8.6 months) of 32(86.5%) patients showed complete occlusion in 23 (71.8%) patients, near-complete occlusion in 6 (18.8%) patients, and incomplete occlusion in 3 (9.4%) patients. Clinical follow-up (mean 33 ± 18.6 months) showed mRS 0–2 in 33 (89.2%) patients and mRS ≥ 3 in 4 (10.8%) patients, including 2 deaths. Large BTAs tended to be a risk factor for procedure-related complications(*p* = 0.08) and unfavorable clinical outcomes(*p* = 0.08).

**Conclusions:**

Traditional coiling and stent-assisted coiling were still the dominant methods for BTAs, supplemented by FD for some complicated conditions such as large/giant or fusiform BTAs. Large size tends to pose additional risks for EVT.

## Background

Basilar trunk artery aneurysms (BTAs) account for 1%–2% of intracranial aneurysms [1–3]. Due to rich perforators and deep anatomical location, the management of BTAs is extraordinary and challenging. Traditional open microsurgery poses great procedure-related complication risks [3, 4]. In recent years, endovascular therapy (EVT), which mainly includes single coiling, low-metal-coverage stent-assisted coiling, and flow diverter (FD) has become the mainstream therapy for BTAs [4–6]. Traditional coiling or stent-assisted coiling could be effective for small and simple saccular BTAs, while it is less effective for complicated conditions such as large, giant, and fusiform BTAs. FD provides an option for neurosurgeons in cases of complicated BTAs. However, the basilar trunk is rich in perforators; thus, it is deemed that the application of FD poses a high risk of ischemic events. Among various endovascular strategies, optimal EVT treatment modalities for patients with BTA are controversial [3, 5, 7]. The knowledge of EVT for BTAs focusing on treatment modalities and complications are inadequate. In this study, we presented our experience of EVT for BTAs through a consecutive series of patients as well as summarized literatures reporting EVT for BTAs.

## Methods

### Patients

**Fig. 1:**
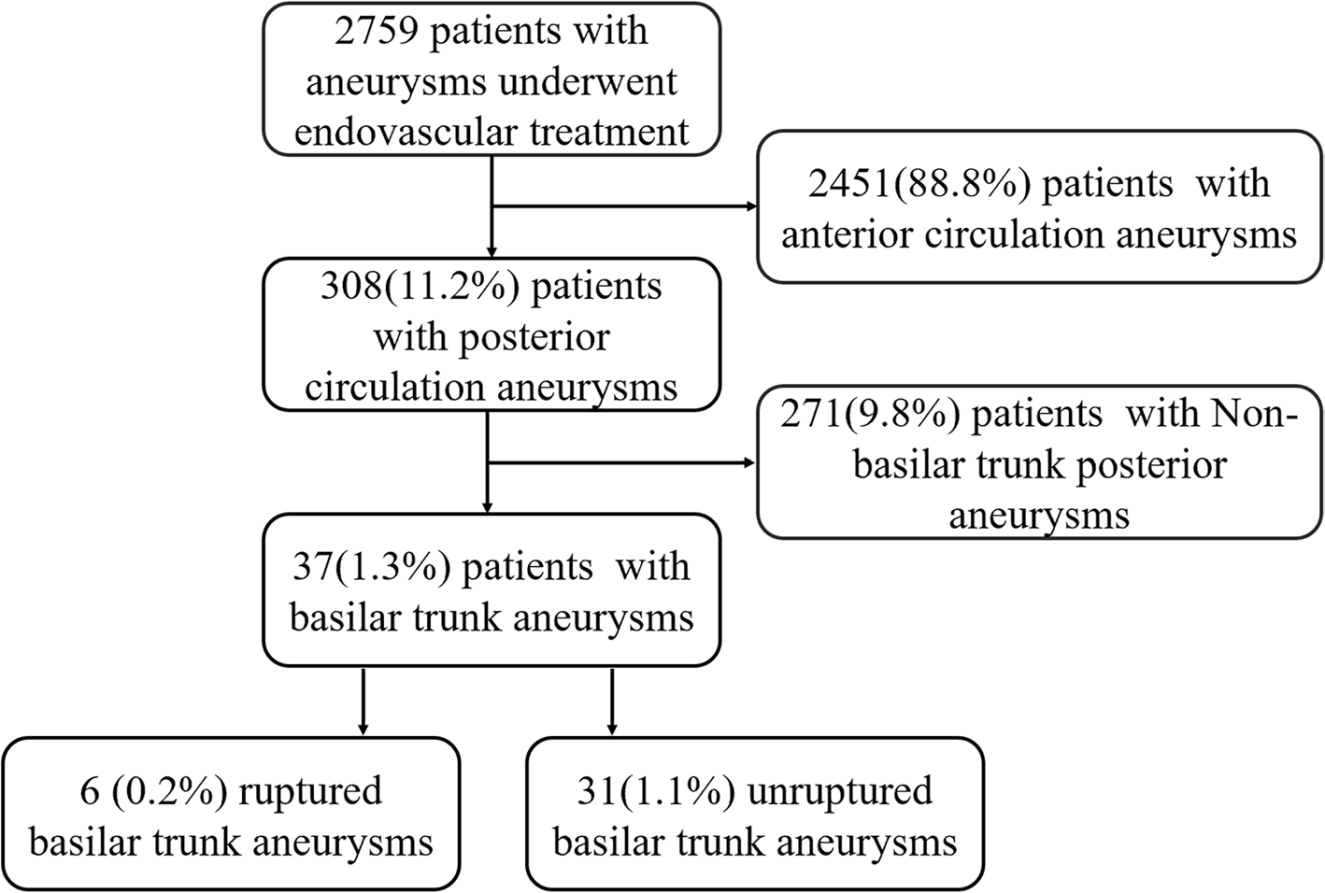
Flow chart of the patient selection process

This study was approved by the Medical Ethics Committee of our institution, and every patient signed informed consent. Patients with BTAs who underwent EVT were retrospectively reviewed from January 2018 to December 2022 at our institution. BTA was defined as aneurysms located distal to the origin of the basilar artery and proximal to the origin of the superior cerebellar artery [3]. A total of 2759 patients were reviewed. Patients with aneurysms located at anterior circulation (*n* = 2451) and non-BTAs (*n* = 271) were excluded. (Fig. 1) Non-basilar trunk posterior aneurysms include vertebrobasilar junction, basilar artery apex, basilar branches (AICA, PICA, and SCA), basilar perforators, and vertebral artery. Patients’ demographics, aneurysm characteristics, treatment modalities, complications and outcomes were collected.

### Definition of variables

The initial presentation was classified as subarachnoid hemorrhage (SAH) confirmed by computed tomography (CT), focal neurological deficit and headache. Some patients had no symptoms and BTA was incidentally found. BTAs are divided into saccular and fusiform aneurysms according to morphology. A saccular aneurysm was defined as an eccentric dilation of the main lumen by a variable-sized neck to an extraluminal pouch. A fusiform aneurysm was defined as a symmetrical or tortuous dilation involving the circumference of the arterial wall [3, 7]. Aneurysm size was measured as the maximal diameter and categorized as small (< 5 mm), medium (5–10 mm), and large (≥ 10 mm).

### Endovascular therapy

Treatment decisions were made by consensus of multi-disciplinary discussion including neurosurgeons, interventional neuroradiologists, and neurologists. Indications for treatment included SAH, focal ischemic symptoms, and a chronological increase in aneurysm size. The choice of techniques (coiling, stent-assisted coiling, or FD) was based on aneurysm size, morphology, parent artery tortuosity, their positional relationship, and expected complications associated with the procedure. Generally, coiling or stent-assisted coiling was the default technique. Simple coiling is selected for small aneurysms (size < 5 mm), saccular morphology, narrow neck, and no risk of coil prolapse. Stent-assisted coiling is selected for wide-neck aneurysms, irregular aneurysm shape, or cases where coil stability is poor during simple coiling. Coiling or stent-assisted coiling is less effective for fusiform, large, or giant aneurysms, or aneurysms for which coiling or stent-assisted coiling is difficult to achieve satisfactory occlusion, for which FD is considered. For unruptured BTAs, dual antiplatelet therapy (daily dose of aspirin 100 mg and clopidogrel 75 mg) was given at least 5 days before the procedure. For ruptured BTAs, a load medication of dual antiplatelet therapy (aspirin 300 mg and clopidogrel 300 mg) was given 4 h before the procedure when a stent was anticipated. EVT was performed under general anesthesia through transfemoral arterial access. A 6- to 8-F sheath was inserted through the femoral artery, and a guiding catheter was navigated into the dominant vertebral artery. The BTA was embolized with coils, stent, stent-assisted coils, or FD according to a standardized routine of our institution. The FD included the Pipeline Embolization Device (Medtronic, USA) and the Tubridge (Microport, China).

### Complications and angiographic and clinical outcome

Procedure-related adverse events include hemorrhagic and ischemic complications. Hemorrhagic complication was defined as a sudden onset of headache, vomiting, and hemorrhage confirmed by CT within 7 days after the procedure. Ischemic complication was defined as any additional neurologic deficits compared with pre-operation and infarctions confirmed by CT/magnetic resonance imaging (MRI) within 7 days after the procedure. Patients were evaluated by digital subtract angiography (DSA) immediately after the procedure and at a 6-month angiographic follow-up after the procedure to confirm the status of aneurysm occlusion. If the aneurysm was still remnant, another DSA would be performed in 1 year. The angiographic results were categorized as complete occlusion, near-complete occlusion (neck remnant), or incomplete occlusion (filling of the aneurysm lumen) according to the Raymond-Roy scale [8]. A favorable angiographic outcome was defined as complete occlusion or neck remnant, and an unfavorable angiographic outcome was defined as incomplete occlusion. All patients received an in-person or telephone clinical outcome follow-up. Clinical outcomes were assessed according to the modified Rankin scale (mRS), where mRS 0–2 is defined as favorable, and mRS ≥ 3 is defined as unfavorable.

### Statistical analysis

Data were presented as means and standard deviations (SD) for continuous variables and proportions for categorical variables. Fisher exact test or Pearson χ^2^ test was used to assess categorical variables. Fisher exact test is used when the expected frequency of any cell in the contingency table is < 5. Pearson χ^2^ test is used when all expected frequencies in the contingency table are ≥ 5. Student’s t-test was used to assess continuous variables. Before use, we performed the Shapiro–Wilk test for normal distribution and Levene's test for homogeneity of variance. All *P* values were 2-sided. Statistical significance was assessed at *p* < 0.05. All statistical analyses were conducted using SPSS 22.0.

## Results

### Demographics, aneurysm characteristics, and treatment details

A total of 37 patients were involved in the study, including 12 (32.4%) females and 25 (67.6%) males. The age ranged from 29 to 77 years old (mean 58.9 ± 8.9 years old). Clinical presentations were SAH (*n* = 6, 16.2%), neurological deficit (*n* = 4, 10.8%), headache (*n* = 11, 29.7%), and no symptoms (*n* = 16, 43.2%). Two (5.4%) patients had multiple intracranial aneurysms (one located at anterior circulation and another one at the basilar trunk, Fig. 1). In unruptured aneurysm cases (*n* = 31), the initial mRS was 0 in 26 patients and 1 in 5 patients. In ruptured aneurysm cases (*n* = 6), initial Hunt and Hess grade (HHG) was 1 in 2 patients, 2 in 2 patients, 3 in 1 patient, and 4 in 1 patient. Clinical poor-grade patients were defined as those with an initial HHG ≥ 4. Nine (24.3%) patients had TIA/ischemic stroke history, and 2 of them received EVT once for stenosis. In terms of morphology, there were 29 (78.4%) saccular and 8 (21.6%) fusiform aneurysms. The mean aneurysm size was 6.6 ± 3.5 mm (range 2.0–17 mm). There were 11 (29.7%) small, 21 (56.8%) medium, and 5 (13.5%) large aneurysms. There were 30 (81.1%) aneurysms with wide necks (neck diameter ≥ 4 mm or dome-to-neck ratio < 2).

Of 37 patients, 5 (13.5%) patients were treated by simple coiling without stent, 1 (2.7%) patient was treated by double low-metal-coverage stent (LVIS, MicroVention, USA) only, 20 (54.1%) patients were treated by stent-assisted coiling, and 11 (29.7%) patients were treated by FDs. Among the 11 patients in whom an FD was used, 4 (36.4%) patients received FD combined with coiling. Double FD was performed in 2 patients. Of 37 patients, immediate postprocedural angiographic outcomes showed complete occlusion in 20 (54.1%) cases, neck remnant in 6 (16.2%) cases, and incomplete occlusion in 11 (29.7%) cases. The frequency of FD use for saccular and fusiform aneurysms was significantly different (5 of 29, 17.2% *vs.* 6 of 8, 75%,* p* = 0.004). The frequency of FD use for small/medium and large aneurysms was also significantly different (7/32, 21.9 *vs.* 4/5, 80%, *p* = 0.021). Patient demographics, aneurysm characteristics, and treatment details are summarized in Table 1.

**Table 1:** Demographics, aneurysm characteristics, and treatment outcomes

Parameters	Value
Number of patients (*n*, %)	37(100.0)
Age (mean ± SD)	58.9 ± 8.9
Gender (*n*, %)	
Female	12(32.4)
Male	25(67.6)
Clinical presentation (*n*, %)	
SAH	6(16.2)
Neurological deficit	4(10.8)
Headache	11(29.7)
Incidental	16(43.2)
Comorbidities (*n*, %)	
Hypertension	27(73)
Diabetes	3(8.1)
TIA/ischemic stroke history	9(24.3)
Aneurysm morphology (*n*, %)	
Fusiform	29(78.4)
Saccular	8(21.6)
Aneurysm size (mean ± SD)	6.6 ± 3.5
Small (*n*, %)	11(29.7)
Medium (*n*, %)	21(56.8)
Large (*n*, %)	5(13.5)
Intervention (*n*, %)	
Coils	5(13.5)
Low-metal-coverage stent	1(2.7)
Coils + stent	20(54.1)
FD	11(29.7)
Complications (*n*, %)	4(10.8)
Hemorrhagic	1(2.7)
Ischemic	3(8.1)
Angiographic outcome (*n*, %)	32(100.0)
Complete occlusion	23(71.8)
Near-complete occlusion	6(18.8)
Incompletely occlusion	3(9.4)
Second operation (*n*, %)	2(5.4)
Follow-up time (month, mean ± SD)	33 ± 18.6
Clinical outcome (*n*, %)	
mRS 0–2	33(89.2)
mRS ≥ 3	4(10.8)

*SAH* subarachnoid hemorrhage, *SD* standard deviation, *FD* flow diverter, *TIA* transient ischemic attack, *mRS* modified Rankin scale

### Complications and angiographic and clinical follow-up

A total of 4 (10.8%) procedure-related complications occurred, including one hemorrhage and 3 ischemia cases. One patient suffered from delayed hemorrhage 2 days after stent-assisted coiling for a large unruptured basilar saccular aneurysm. The patient died 4 days after delayed hemorrhage. Two patients developed hemiplegia due to perforator ischemia. One suffered from right upper limb weakness several hours after stent-assisted coiling for an unruptured saccular aneurysm. The mRS was 1 at a 35-month follow-up. The other one suffered from right limb weakness and cough 5 days after simple coiling for a ruptured saccular aneurysm. The mRS was 3 at a 32-month follow-up. One patient developed limb weakness and aphasia 1 day after double FD for a large fusiform aneurysm, which was due to in-stent thrombosis. The mRS was 4 at a 24-month follow-up. Tirofiban, volume expansion, anti-vasospasm treatment, and agents promoting collateral circulation were adopted for ischemic complication patients.

**Fig. 2:**
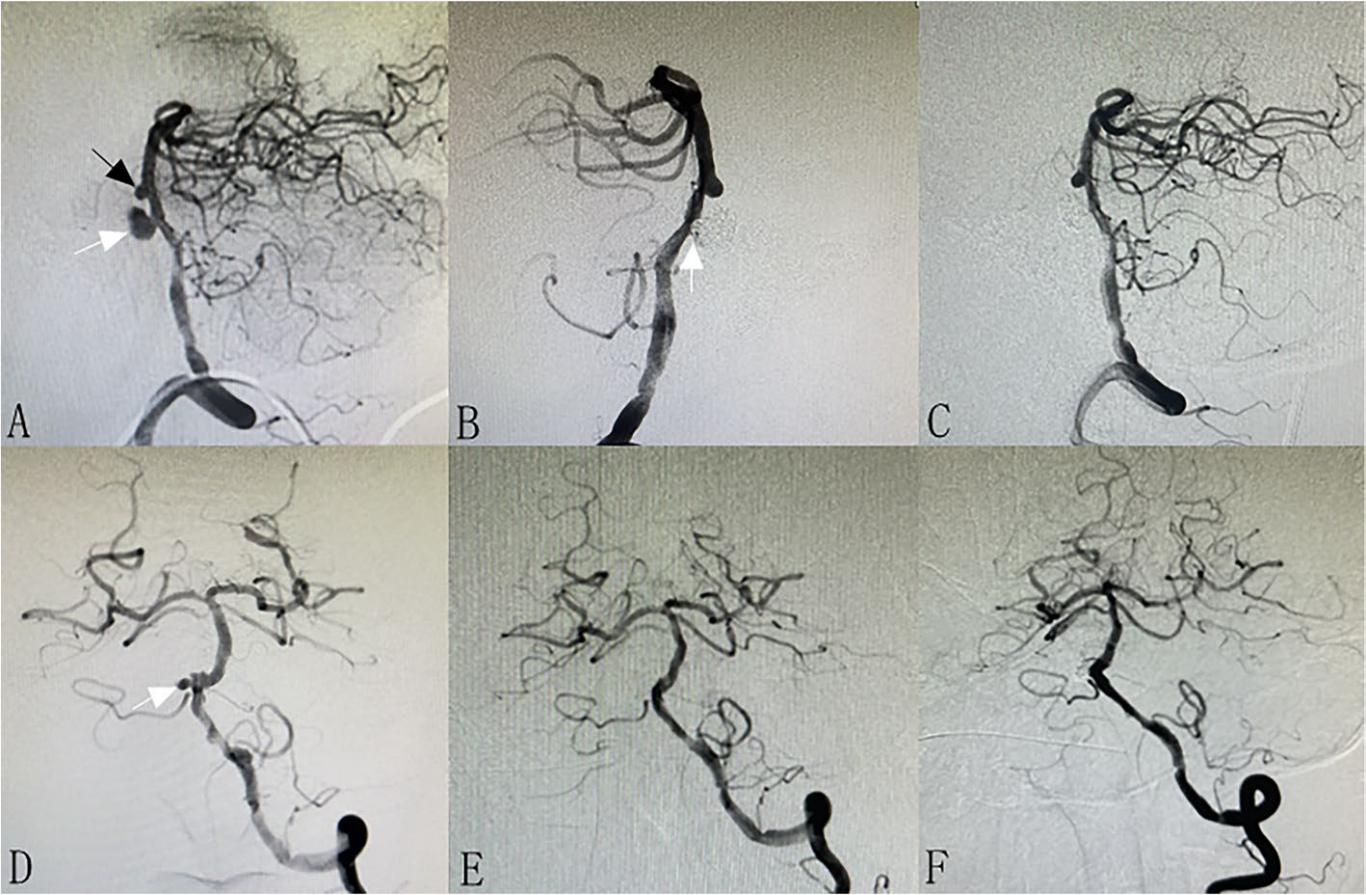
A patient with two unruptured BTAs (**A**, arrows). Stent-assisted coiling was performed for the bigger BTA, and the small aneurysm was left for observation. Immediate post-operative angiography showed neck remnant (**B**, arrow). Six-month follow-up angiography showed complete occlusion of the BTA. The small aneurysm did not change in size and morphology (**C**). A patient with a ruptured BTA (**D**, arrow). Stent-assisted coiling was performed for the BTA, and immediate post-operative angiography showed complete occlusion (**E**). Six-month follow-up angiography showed complete occlusion and no recanalization (**F**)

**Fig. 3:**
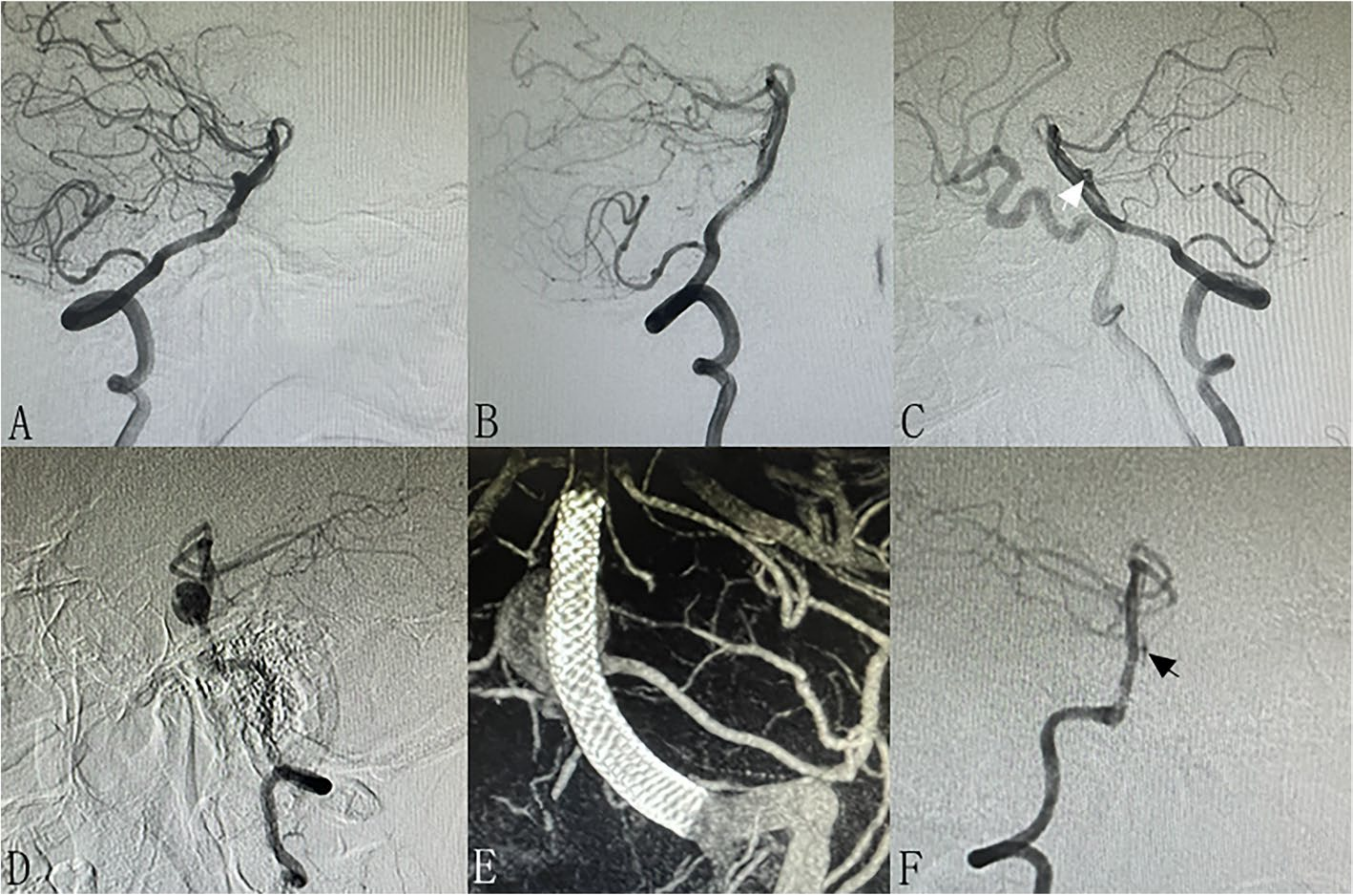
A patient with a ruptured BTA (**A**). Stent-assisted coiling was performed for the BTA, and immediate post-operative angiography showed neck remnant (**B**). One-year follow-up angiography showed neck remnant (**C**, arrow). A patient with an unruptured BTA (**D**). FD (Pipeline, Medtronic, USA) was used for the BTA, and dynamic CT showed the stent (**E**). One-year follow-up angiography showed neck remnant (**F**, arrow)

**Fig. 4:**
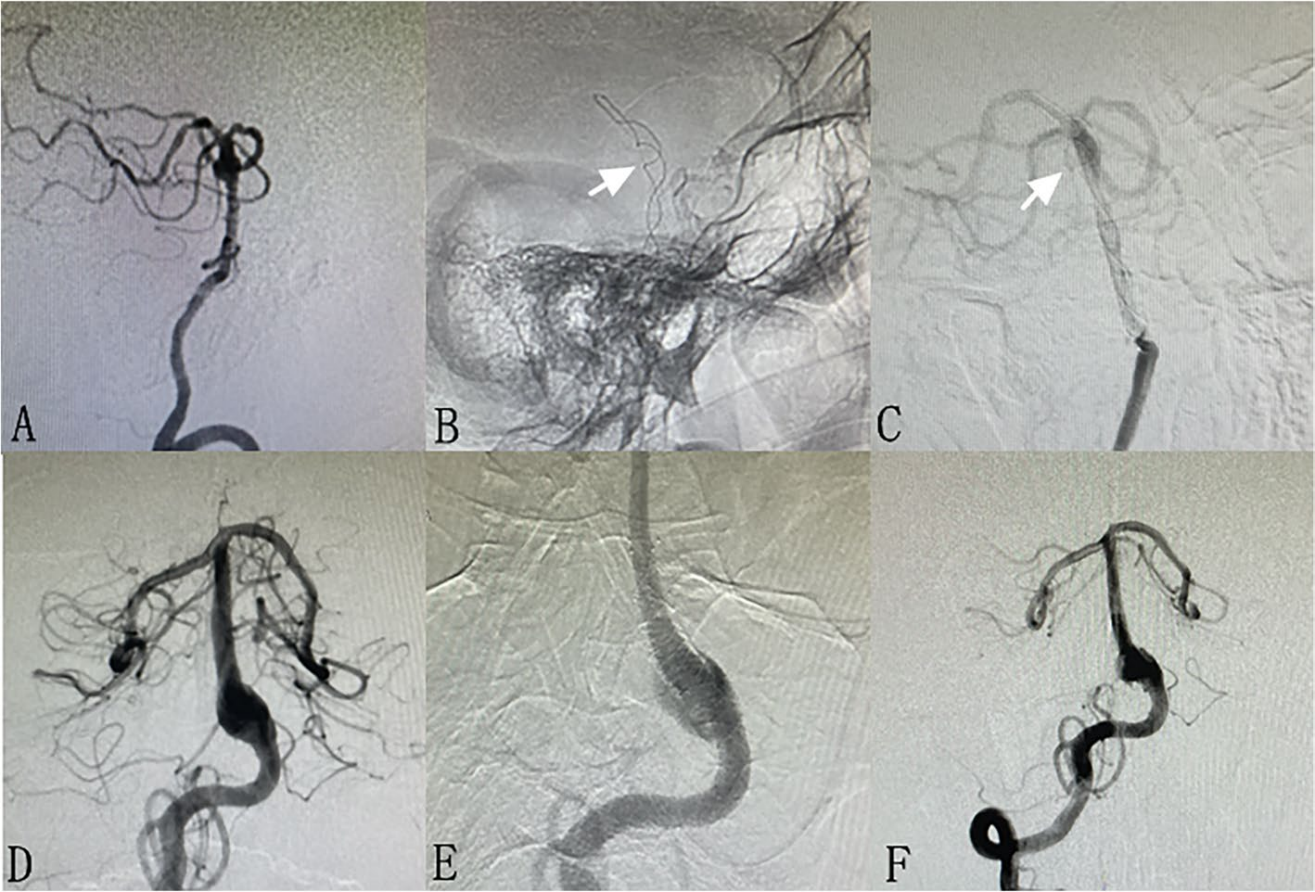
A patient with an unruptured BTA (**A**). FD (Tubridge, Microport, China) was used for the BTA, and non-subtraction angiography showed the stent (**B**, arrow). One-year follow-up angiography showed very slight lumen filling of contrast agent (**C**, arrow). A patient with an unruptured BTA (**D**). A double traditional low metal coverage stent (LVIS, MicroVention, USA) was used for the BTA, and post-operative angiography showed the stent (**E**). One-year follow-up angiography showed lumen filling of contrast agent (**F**)

Thirty-two (86.4%) patients received angiographic follow-up. The mean angiographic follow-up time was 9.5 ± 8.6 months. Five patients were absent, including 2 dead patients and 3 patients who were lost to follow-up. Among the 32 patients, the last angiographic follow-up results showed complete occlusion in 23 (71.8%) patients (Fig. 2), neck remnant in 6 (18.8%) patients (Fig. 3), and incomplete occlusion in 3 (9.4%) patients (Fig. 4). Overall, favorable angiographic outcomes (complete occlusion or neck remnant) were achieved in 29 (90.6%) patients.

Clinical follow-up was performed for all patients. A mean 33 ± 18.6-months clinical follow-up showed mRS 0–2 in 33 (89.2%) patients and mRS ≥ 3 in 4 (10.8%) patients. Of the six patients with ruptured BTA, one died from initial hemorrhage (initial Hunt-Hess grade = 4). One patient experienced a complication presented as right limb weakness and cough 5 days after simple coiling for a saccular aneurysm which is due to perforator ischemia. The mRS was 3 at a 32-month follow-up. The mRS of the other 4 patients was 0 in 2 patients and 1 in 2 patients. Of the 31 patients with unruptured BTAs, 3 experienced procedure-related complications, including 2 ischemia and 1 hemorrhage. The mRS was 1, 4, and 6, respectively. In total, favorable clinical outcome was achieved in 33 (89.2%) patients, including 29 (93.5%) patients with unruptured aneurysms and 4 (66.7%) patients with ruptured aneurysms. Unfavorable clinical outcomes occurred in 4 (10.8%) patients, including 2 (6.5%) patients with unruptured aneurysms and 2 (33.3%) patients with ruptured aneurysms, and 2 (5.4% of total) patients who died. A comparison of patients with and without complications revealed that large aneurysm size tended to be a risk factor (*p* = 0.08) (Table 2). No factor associated with complete and incomplete occlusion was found (Table 3). Large aneurysm size tended to be a risk factor for unfavorable clinical outcomes (*p* = 0.08) (Table 4).

**Table 2:** Clinical predictors of procedure-related complications

	Complication *n* = 4	Non-complication *n* = 33	*P*-value
Female (*n*, %)	2(50)	10(30.3)	0.582
Age (mean ± SD)	61.8 ± 8.3	58.5 ± 9.0	0.500
Hemorrhage presentation (*n*, %)	1(25)	5(15.2)	0.524
TIA/stroke history (*n*, %)	0(0)	9(27.3)	0.554
Hypertension (*n*, %)	2(50)	25(75.8)	0.291
Diabetes (*n*, %)	1(25)	2(6.1)	0.298
TIA/stroke history (*n*, %)	0(0)	9(27.3)	0.554
Morphology (*n*, %)			1.000
Saccular	3(75)	26(78.8)	
Fusiform	1(25)	7(21.2)	
Large/Giant (*n*, %)	2(50)	3(9.1)	0.080
Wide neck (*n*, %)	3(75)	27(81.8)	1.000
Intervention (*n*, %)			1.000
Coils/stent/coils + stent	3(75)	23(69.7)	
FD	1(25)	10(30.3)	

*SD* standard deviation, *mRS* modified Rankin scale, *FD* flow diverter, *TIA* transient ischemic attack

**Table 3:** Angiographic outcome predictors of complete occlusion

	Complete occlusion *n* = 23	Near-complete and Incomplete occlusion *n* = 9	*P*-value
Female (*n*, %)	6(26.1)	3(33.3)	0.685
Age (mean ± SD)	58.7 ± 6.5	58.7 ± 13.4	0.984
Hemorrhage presentation (*n*, %)	2(8.7)	2(22.2)	0.557
Hypertension (*n*, %)	16(69.6)	7(77.8)	1.000
Diabetes (*n*, %)	1(4.3)	1(11.1)	0.490
TIA/stroke history (*n*, %)	8(34.8)	1(11.1)	0.383
Morphology (*n*, %)			0.314
Saccular	20(87.0)	6(13.0)	
Fusiform	3(13.0)	3(33.3)	
Aneurysm size (mean ± SD)	6.4 ± 3.7	6.3 ± 1.5	0.981
Wide neck (*n*, %)	18(78.3)	8(88.9)	0.648
Large (*n*, %)	1(4.3)	2(22.2)	0.184
Intervention (*n*, %)			0.407
Coils/stent/coils + stent	17(73.9)	5(55.6)	
FD	6(26.1)	4(44.4)	
Follow-up time (month, mean ± SD)	35.6 ± 17.1	26.8 ± 18.8	0.213

*SD* standard deviation, *mRS* modified Rankin scale, *FD* flow diverter, *TIA* transient ischemic attack

**Table 4:** Favorable and unfavorable clinical outcome predictors

	Total patients 37	Favorable clinical outcome *n* = 33	Unfavorable clinical outcome *n* = 4	*P*-value
Female (*n*, %)	12(32.4)	9(27.3)	3(75)	0.091
Age (mean ± SD)	58.9 ± 8.9	58.9 ± 8.6	58.7 ± 12.3	0.979
Hemorrhage presentation (*n*, %)	6(16.2)	4(12.1)	2(50)	0.115
Morphology (*n*, %)				0.640
Saccular	29(78.4)	26(78.8)	3(75)	
Fusiform	8(21.6)	7(21.2)	1(25)	
Large/giant (*n*, %)	5(13.5)	3(9.1)	2(50)	0.080
Intervention (*n*, %)				
Coils/coils + stent	26(70.3)	23(69.7)	3(75)	1.000
FD	11(29.7)	10(30.3)	1(25)	

*SD* standard deviation, *FD* flow diverter

## Discussion

BTAs have diverse clinical and angiographic features and are difficult to treat, requiring various techniques and multiple procedures [7]. Neurosurgical treatment of BTAs is challenging due to the narrow surgical corridor and the proximity of cranial nerves, perforating arteries, and brain stem [3]. As a result, endovascular techniques have become the mainstay of treatment [9–11]. We reviewed literatures from 2013 to 2024 reporting EVT for BTA. Studies with more than 5 cases were included. A total of 7 studies were involved, including 242 cases [3, 5–7, 12–14]. (Table 5) A total of 98 cases were ruptured, accounting for 40.5%. Coils with or without stent were used for 217(89.7%) cases and FD were used for 25(10.3%) cases. A total of 58(23.9%) procedural related complications happened, including 52(21.5%) ischemic events and 6(2.4%) hemorrhagic events. In all, 176(72.7%) cases received angiographic follow and 134(76.1%) showed favorable. Clinical follow-up showed that 185(76.4%) cases recovered well. The homogeneity among studies is inadequate. For example, Sook YS reported 2 studies about BTA [7, 13], one reported 27 BTA patients and all of them are ruptured, another reported 40 patients and 20(50%) are large or giant BTA. Majority of BTA patients were treated by coils or stent assisted coils. Larger aneurysms and high preoperative Hunt-Hess grade may predict unfavorable clinical outcome [7, 14].

**Table 5:** Literature review of EVT for BTA

Authors/ Year	No. of patients	Large or Giant, n(%)	Ruptured n(%)	Stent + coils or coils/FD	Ishchemic/Hemo Complication	Favorable Angiographic Outcome (n, %)	Favorable Clinical outcome (n, %)
Van et al., 2013 [12]	13	11(84.6)	3(23.1)	11/2	3/1	9(69.2)	9(69.2)
Saliou G et al., 2015 [3]	8	NA	5(62.5)	7/1	1/0	6(75.0)	5(62.5)
Cho KC etal., 2019 [6]	15	2(13.3)	7(46.7)	15/0	1/0	11(73.3)	12(80.0)
Sim SY et al., 2022 [7]	40	20(50.0)	27(67.5)	35/5	5/0	26(83.9) of 31	27(67.5)
Wang et al., 2021 [5]	28	8(28.6)	10(35.7)	28/0	9/1	9(81.8) of 11	23(82.1)
Zhong et al., 2023 [14]	111	NA	26(6.1)	97/14	29/4	57(74.0) of 77	92(83.3)
Sim SY et al., 2023 [13]	27	13(48.1)	20(100)	24/3	4/0	16(76.2) of 21	17(64.0)
Sub-Total	242	54(43.9)	98(40.5)	217/25	52/6	134(76.1) of 176	185(76.4)
This study	37	5(13.5)	6(16.2)	26/11	3/1	29(90.6) of 32	33(89.2)
Total	279	59(21.1)	104(37.3)	243/36	55/7	163(78.4) of 208	218(78.1)

*FD* flow diverter, *Hemo. complication* Hemorrhagic complication, *NA* not available

In this report, we reviewed our outcomes with EVT of BTAs. A total of 37/2759(1.3%) patients were involved, including 6(16.2%) patients with ruptured aneurysms and 31(83.8%) patients with unruptured aneurysms. Twenty-six patients received traditional simple coiling or stent-assisted coiling, and 11 patients received FD implantation. A total of 4 (10.8%) patients experienced procedure-related complications. Angiographic and clinical follow-up results revealed complete or near-completely occlusion in 29/32 (90.6%) patients and favorable clinical outcomes in 33 (89.2%) patients. The result confirmed that endovascular therapy for BTA was safe and effective, with acceptable morbidity and mortality given its complexity. Large aneurysm size was a potential risk factor for perioperative complications, which needs to be confirmed by a large cohort.

In terms of treatment modalities, coiling/stent-assisted coiling and FD are both optional for most BTAs. Traditional stent-assisted coiling often has high porosity and cannot function as FDs, while FDs pose a higher risk of basilar perforator occlusion [15, 16]. Peng et al. have reviewed 80 patients with basilar trunk and vertebrobasilar junction aneurysms, including 58 BTAs. They concluded that FDs or conventional stents were both feasible and effective for small BTVBJ aneurysms. For patients with large or giant aneurysms, treatment using FDs achieved higher rates of occlusion and favorable clinical outcomes than conventional stent-assisted coiling [17]. In this study, one patient was treated by double low-metal-coverage stent (LVIS, MicroVention, USA) only. This patient had an unruptured wide-neck fusiform aneurysm. The aneurysm neck involved the origin of the anterior inferior cerebellar artery (AICA), and single stent could not cover both the aneurysm neck and AICA origin. Preoperative simulation showed that double overlapping stents (LVIS) could reconstruct the vascular wall and prevent coil prolapse without occluding AICA. The patient refused FD treatment due to concerns about long-term antiplatelet therapy, so double LVIS stents were selected. In this cohort, a total of 11 BTAs were treated with FD. Double FDs were used in 2 cases, both of which were large fusiform aneurysms involves a long segment of the basilar trunk. Single FD cannot fully cover the aneurysm neck and proximal/distal normal vessels. High risk of residual aneurysm lumen after single FD implantation. The frequency of FD use for fusiform aneurysms was significantly higher than that of saccular aneurysms (5 of 29, 17.2% *vs.* 6 of 8, 75%, *p* = 0.004). The frequency of FD use for large aneurysms was also significantly higher than that of small/medium aneurysms (4/5, 80% *vs.* 7/32, 21.9%, *p* = 0.021). The efficacy of FD for large/fusiform aneurysms is higher than that of traditional coiling/stent-assisted coiling. No difference was noted in clinical or angiographic outcomes among various treatment techniques. The FD adoption rate (29.7%) of this cohort was higher than that of the literature average (10.3%), which is related to high percentage of fusiform aneurysm (78.4%), for which coiling or stent-assisted coiling is less effective. When treating BTAs, traditional coiling/stent-assisted coiling and FD are complementary rather than competing techniques [3].

The BA trunk contains many perforators supplying the brainstem. Furthermore, the perforators of the BA are tiny and important, resulting in a high ischemic complication rate, especially when FD is adopted [5]. A retrospective study of 28 patients has been performed for BTA patients who underwent EVT. The patients were given single coiling or stent-assisted coiling. Among the 28 patients, 9 patients experienced ischemic complications, with an incidence of 32.1%. All nine incidents of ischemic complications were caused by perforator occlusion or BA ischemia [5]. In this study, coiling or stent-assisted coiling was the default technique, and FD would be considered when confronting large/giant or fusiform BTAs to avoid a high rate of ischemia. A total of 4 complications occurred, including 3 (75%) ischemia cases. The procedure-related complication rate in this study was not significantly different between the FD (1/11, 9.1%) group and the conventional stent group (3/26, 11.5%). Large size tended to be a factor associated with complications, which is inclined with previous literatures. This might be due to the involvement of more perforators, stem mass effect, detachment of thrombus, and long operating time. Ischemic complications deserve our attention for their high incidence and poor clinical outcome despite treatment modalities [14].

BTAs are more often dissection aneurysms rather than saccular bifurcation aneurysms [18]. The overall recanalization rates of EVT of BTAs have been reported as 20.9%–33.3%, and good clinical outcomes have been reported in 78.6% of patients [6]. Sim et al. have reviewed EVT for 40 BTAs. Favorable clinical and angiographic outcomes were achieved in 65.0% and 83.9% of patients, respectively. The reason for the high recurrence rates might be that most BA trunk aneurysms are large or giant in size with a wide neck or fusiform configuration [7]. In this study, the last angiographic results showed complete occlusion in 71.8% of cases, near-complete occlusion in 18.8% of cases, and incomplete occlusion in 9.4% of cases. Favorable angiographic outcomes were achieved in 90.6% of patients. The favorable clinical outcome rate was 89.2%. Both the occlusion rate and favorable clinical outcome rate were higher in this study compared with the reported literatures. The reason might be the lower rate of patients with ruptured (98/242, 40.5% *vs.* 6/37, 16.2%) and large/giant BTAs (54/123, 43.9% *vs.* 5/37, 13.5%), which may lead to overestimated favorable outcomes. (Table 5) Factors associated with complication and complete occlusion were important for the treatment modality option.

### Limitations

This was a retrospective study. This series only included patients who were treated, and the outcomes for those followed conservatively were not reported. This represents a selection bias, of probably preferentially selecting for aneurysms that could be treated with lower risks. The treatment of fusiform and saccular BTAs is different. The small cohort limits subgroup comparisons (e.g., FD vs. stent outcomes). Only 11 patients received FDs and only 8 fusiform aneurysms were included, which may limit the generalization of results to these subgroups. The FD group (*n* = 11) and stent group (*n* = 21) had small sample sizes, leading to insufficient statistical power for outcome comparison. Even that there is no difference in terms of completely occlusion, complications and unfavorable clinical outcomes between fusiform and saccular BTAs, it is better to analysis them separately, which is limited by small sample of this study. Large cohort studies are needed.

## Conclusions

Traditional coiling and stent-assisted coiling were still the dominant methods for BTAs, supplemented by FD for some complicated conditions such as large/giant or fusiform BTAs. Large size tends to pose additional risks for EVT.

## Data Availability

The datasets used and/or analyzed during the current study are available from the corresponding author on reasonable request.

## Abbreviations

BTA: Basilar trunk aneurysm
EVT: Endovascular treatment
FD: Flow diverter
SAH: Subarachnoid hemorrhage
